# Real‐World Assessment of Trilaciclib for the Prevention of Chemoradiotherapy‐Induced Myelosuppression in Esophageal Squamous Cell Carcinoma: A Propensity Score Matching Study

**DOI:** 10.1002/cam4.70862

**Published:** 2025-04-15

**Authors:** Jingze Yan, Zeyuan Liu, Hui Chen, Xinchen Sun, Xiaolin Ge, Xiaojie Xia

**Affiliations:** ^1^ Department of Radiation Oncology The First Affiliated Hospital of Nanjing Medical University Nanjing China; ^2^ Department of Radiation Oncology The Affiliated Jiangning Hospital of Nanjing Medical University Nanjing China; ^3^ Department of Oncology Kangda College of Nanjing Medical University Lianyungang China

**Keywords:** CDK4/6 inhibitor, concurrent chemoradiotherapy, esophageal cancer, myelosuppression, trilaciclib

## Abstract

**Background:**

Chemoradiotherapy‐induced myelosuppression (CIM) is the most common adverse event of esophageal cancer treatment, often necessitating reductions or delays in chemotherapy. Current treatments target specific blood cells, causing adverse effects. Trilaciclib, a novel CDK4/6 inhibitor with myeloprotective effects, has not yet been evaluated for its use in esophageal cancer treatment. We aimed to investigate the efficacy and safety of trilaciclib in preventing CIM.

**Methods:**

Clinical data were retrospectively collected from 203 patients with esophageal cancer who underwent concurrent radiotherapy at the Department of Radiotherapy of Jiangsu Province People's Hospital between January 2022 and January 2024. Patients were divided into the trilaciclib group (34 patients) and control group (169 patients). Propensity score matching (PSM) was performed to balance the baseline characteristics, and the incidence of myelosuppression and adverse events was compared.

**Results:**

Following PSM, 34 patients were included in each group, with no significant differences in baseline characteristics. The trilaciclib group exhibited significantly higher leukocyte, neutrophil, hemoglobin, and platelet levels (*p* < 0.05). The trilaciclib group exhibited a lower incidence of grade III–IV neutropenia and leukopenia, and none developed febrile neutropenia. Objective remission and disease control rates were comparable between the groups, with 1‐year overall survival and progression‐free survival rates of 82.0% and 73.4% in the trilaciclib group and 78.9% and 72.7% in the control group (not significant). The incidence of non‐hematological toxic events was similar between the groups (*p* > 0.05).

**Conclusion:**

Trilaciclib prevented myelosuppression in patients with esophageal cancer undergoing concurrent chemoradiotherapy, demonstrating good safety and efficacy.

## 1 Introduction

Esophageal cancer (EC) is a prevalent digestive system cancer with a poor prognosis. It currently ranks seventh in incidence and sixth in mortality among all cancer types, with 604,000 new cases per year and 544,000 deaths annually worldwide. More than half of the new cases and deaths occurred in China [1, 2].

Symptoms of early‐stage EC are often subtle and difficult to detect. Consequently, many patients are already in the middle or late stages of the disease by the time they seek medical treatment, thus losing the opportunity for radical surgery. Synchronous chemoradiotherapy is the preferred treatment option for patients with EC who are not candidates for radical surgery [3]. Although concurrent chemoradiotherapy improves tumor control and prolongs patient survival, it is also associated with significant toxic side effects, such as bone marrow suppression and liver and kidney damage [4, 5]. Myelosuppression is the most common dose‐limiting side effect of chemotherapy. A significant number of patients must reduce the chemotherapy dose or discontinue treatment due to chemotherapy‐induced myelosuppression, which negatively impacts antitumor efficacy [6]. Myelosuppression primarily manifests as neutropenia, anemia, and thrombocytopenia. It is typically treated with growth factors, which target only a single blood cell lineage, require prolonged treatment, and produce slow effects. Adverse reactions related to the drugs and blood transfusions pose additional risks. Repeated mobilization of bone marrow hematopoietic stem cells can also lead to bone marrow exhaustion [7].

Trilaciclib is a highly effective, reversible, and innovative CDK4/6 inhibitor. It is the first drug worldwide to provide full‐lineage bone marrow protection. Administering trilaciclib prior to chemotherapy temporarily halts the division of bone marrow cells, protecting them from the toxicity of chemotherapeutic agents and preventing bone marrow depletion, thereby preserving hematopoietic function without affecting the antitumor efficacy of chemotherapy [8]. Trilaciclib was approved by the US Food and Drug Administration in February 2021 to reduce the incidence of bone marrow suppression in adult patients undergoing chemotherapy. It was approved for marketing in China in July 2022 and is currently indicated for the treatment of extensive‐stage small cell lung cancer (SCLC) [9]. However, no clinical research has examined whether trilaciclib can prevent bone marrow suppression during concurrent chemoradiotherapy for EC. Therefore, this study is the first to explore the clinical efficacy and safety of trilaciclib in preventing bone marrow suppression during concurrent chemoradiotherapy for EC, thereby providing evidence‐based support for its application in patients with this condition.

## 2 Materials and Methods

### 2.1 Patients

This retrospective analysis included 203 patients with esophageal squamous carcinoma who received concurrent chemoradiotherapy with or without trilaciclib treatment at the Department of Radiotherapy of Jiangsu Province People's Hospital between January 2022 and January 2024. Patients (1) aged ≥ 18 years, (2) with a pathological diagnosis of esophageal squamous cell carcinoma, (3) who were unable to undergo surgical treatment, and (4) who completed definitive chemoradiotherapy or chemoradiotherapy combined with immunotherapy were included. Meanwhile, patients who had (1) a history of other malignant tumors, (2) difficulties with follow‐up, and (3) incomplete clinical information were excluded. Clinical staging was determined based on the 8th edition of the Union for International Cancer Control. This study was approved by the Research Ethics Committee of the First Affiliated Hospital of Nanjing Medical University. Written informed consent was obtained from all patients, who consented to the use of their clinical data for research purposes.

### 2.2 Treatment Schedules

In this study, all patients received definitive chemoradiotherapy or chemoradiotherapy combined with immunotherapy. Intensity‐modulated radiation therapy was used, delivering a total radiation dose of 50–50.4 Gy (in 1.8–2.0 Gy fractions). Patients received a chemotherapy regimen consisting of paclitaxel (135 mg/m^2^ Day 1) and nedaplatin (75 mg/m^2^ Days 1–2) administered every 3 weeks. The immunotherapy regimen included an anti‐PD‐1 antibody, administered every 3 weeks. Clinicians selected an individualized regimen based on the patient's condition and preferences. After completion of radiotherapy, the patients received two cycles of consolidation treatment with the same regimen. Trilaciclib, a CDK4/6 inhibitor with a myeloprotective indication, was administered pre‐chemotherapy. Patients in the trilaciclib group received intravenous trilaciclib (240 mg/m^2^) 4 h prior to the administration of chemotherapy, whereas the control group did not. Long‐ or short‐acting granulocyte colony‐stimulating factors (G‐CSFs) were administered in both groups according to the National Comprehensive Cancer Network guidelines on hematopoietic growth factors, following the development of myelosuppression during treatment.

### 2.3 Endpoints

The primary endpoint was the incidence of myelosuppression during treatment, which commonly manifests as leukopenia, neutropenia, anemia, and thrombocytopenia. The key secondary endpoints were febrile neutropenia (FN) and other systemic adverse events. FN was defined as a fever of 38.3°C or higher or a sustained temperature of 38.0°C for more than 1 h in neutropenic patients with a neutrophil count of less than 500 cells/μL [10]. Myelosuppression and other systemic adverse events were evaluated based on the National Cancer Institute Common Terminology Criteria for Adverse Events version 5.0. Other endpoints included white blood cell count, neutrophil count, lymphocyte count, hemoglobin level, platelet count, antibiotic use, tumor response, progression‐free survival (PFS), and overall survival (OS). The response of solid tumors was classified as complete response (CR), partial response (PR), stable disease (SD), or progressive disease (PD), in accordance with the revised Response Evaluation Criteria in Solid Tumors (version 1.1) [11]. In addition, the objective response rate (ORR) was defined as the sum of CR and PR, whereas the disease control rate (DCR) was defined as the sum of CR, PR, and SD. Three months after the completion of concurrent chemoradiotherapy, imaging examinations were performed to evaluate short‐term efficacy. PFS was calculated as the time interval from the date of treatment to the date of progression, relapse, death, or last contact. OS was calculated as the time interval from the date of treatment to the date of death or last contact.

### 2.4 Propensity Score Analysis

To reduce the bias resulting from non‐randomized allocation, propensity score matching (PSM) was employed to balance the baseline characteristics between the trilaciclib and control groups. PSM was performed using a logistic regression model that included variables such as sex, age, body mass index (BMI), smoking, alcohol consumption, nutritional management mode, performance status (PS) score, clinical stage, grade, tumor size, tumor location, and treatment plan. Patients were matched in a 1:1 ratio using a nearest‐neighbor matching algorithm. A caliper of 0.2 was applied as the maximum tolerated difference. PSM was conducted using Statistical Package for the Social Sciences (SPSS) version 22.0 software (SPSS Inc., Chicago, IL, USA).

### 2.5 Statistical Analysis

Patient characteristics, adverse events, and tumor responses were analyzed using Pearson's chi‐square or Fisher's exact test. Continuous variables were expressed as the medians (interquartile range). Survival curves were generated using the Kaplan–Meier method, with comparisons made using the log‐rank test. All statistical tests were two‐tailed, and a *p* value of < 0.05 (95% confidence interval) was considered significant. All statistical analyses were performed using SPSS version 22 software (IBM Corp., Armonk, NY, USA).

## 3 Results

### 3.1 Patients

We retrospectively reviewed 203 patients (34 in the trilaciclib group and 169 in the control group). Prior to matching, the baseline clinical characteristics between the two groups were not comparable. Therefore, a 1:1 PSM analysis was conducted to balance these characteristics. After PSM, each group comprised 34 patients. No significant differences were observed between the two groups in terms of age, sex, BMI, PS score, dietary status, smoking and drinking history, tumor stage, degree of tumor differentiation, treatment therapy, tumor length, and location (Table 1, all *p* > 0.05).

**TABLE 1:** Baseline patient characteristics before and after propensity score matching.

	Total (*n* = 203)			PSM (1:1) (*n* = 68)		
	Control group (*n* = 169)	Trilaciclib group (*n* = 34)	*p*	Control group (*n* = 34)	Trilaciclib group (*n* = 34)	*p*
Gender			0.774			0.779
Male	133 (78.7%)	26 (76.5%)		25 (73.5%)	26 (76.5%)	
Female	36 (21.3%)	8 (23.5%)		9 (26.5%)	8 (23.5%)	
Age			0.431			0.417
< 65	30 (17.8%)	8 (23.5%)		11 (32.4%)	8 (23.5%)	
≥ 65	139 (82.2%)	26 (76.5%)		23 (67.6%)	26 (76.5%)	
BMI			0.898			0.418
< 18.5	24 (14.2%)	4 (11.8%)		7 (20.6%)	4 (11.8%)	
18.5–24	88 (52.1%)	19 (55.9%)		14 (41.2%)	19 (55.9%)	
≥ 24	57 (33.7%)	11 (32.4%)		13 (38.2%)	11 (32.4%)	
Smoking			0.828			0.808
Yes	78 (46.2%)	15 (44.1%)		16 (47.1%)	15 (44.1%)	
No	91 (53.8%)	19 (55.9%)		18 (52.9%)	19 (55.9%)	
Alcohol abuse			0.972			0.808
Yes	74 (43.8%)	15 (44.1%)		16 (47.1%)	15 (44.1%)	
No	95 (56.2%)	19 (55.9%)		18 (52.9%)	19 (55.9%)	
Nutritional management mode			0.816			0.734
Soft diet	38 (22.5%)	7 (20.6%)		8 (23.5%)	7 (20.6%)	
Semi‐liquid diet	102 (60.4%)	22 (64.7%)		19 (55.9%)	22 (64.7%)	
Liquid diet	25 (14.8%)	5 (14.7%)		7 (20.6%)	5 (14.7%)	
Naso‐intestinal tube/gastrostomy tube	4 (2.4%)	0		0	0	
PS socre			0.049			0.742
0	59 (34.9%)	6 (17.6%)		5 (14.7%)	6 (17.6%)	
1	110 (65.1%)	28 (82.4%)		29 (85.3%)	28 (82.4%)	
Clinical stage			0.551			1.000
I	4 (2.4%)	0		0	0	
II	55 (32.5%)	11 (32.4%)		11 (32.4%)	11 (32.4%)	
III	71 (42.0%)	12 (35.3%)		12 (35.3%)	12 (35.3%)	
IV	39 (23.1%)	11 (32.4%)		11 (32.4%)	11 (32.4%)	
Grade			0.536			1.000
G1	13 (7.7%)	1 (2.9%)		1 (2.9%)	1 (2.9%)	
G2	97 (57.4%)	19 (55.9%)		19 (55.9%)	19 (55.9%)	
G3	59 (34.9%)	14 (41.2%)		14 (41.2%)	14 (41.2%)	
Tumor size			0.237			0.804
< 5 cm	52 (30.8%)	14 (41.2%)		13 (38.2%)	14 (41.2%)	
≥ 5 cm	117 (69.2%)	20 (58.8%)		21 (61.8%)	20 (58.8%)	
Tumor location			0.044			0.579
Cervical	7 (4.1%)	6 (17.8%)		3 (8.8%)	6 (17.6%)	
Upper chest	37 (21.9%)	4 (11.8%)		8 (23.5%)	4 (11.8%)	
Middle chest	51 (30.2%)	9 (26.5%)		11 (32.4%)	9 (26.5%)	
Lower chest	58 (34.3%)	11 (32.4%)		9 (26.5%)	11 (32.4%)	
Overlapping	16 (9.5%)	4 (11.8%)		3 (8.8%)	4 (11.8%)	
Treatment plan			< 0.001			0.720
CRT	89 (52.7%)	4 (11.8%)		5 (14.7%)	4 (11.8%)	
CRT combined with immunotherapy	80 (47.3%)	30 (88.2%)		29 (85.3%)	30 (88.2%)	

Abbreviations: BMI, body mass index (calculated as weight in kilograms divided by height in meters squared); CRT, chemoradiotherapy; PS, performance status.

### 3.2 Hematological Toxicity Reactions

**FIGURE 1:**
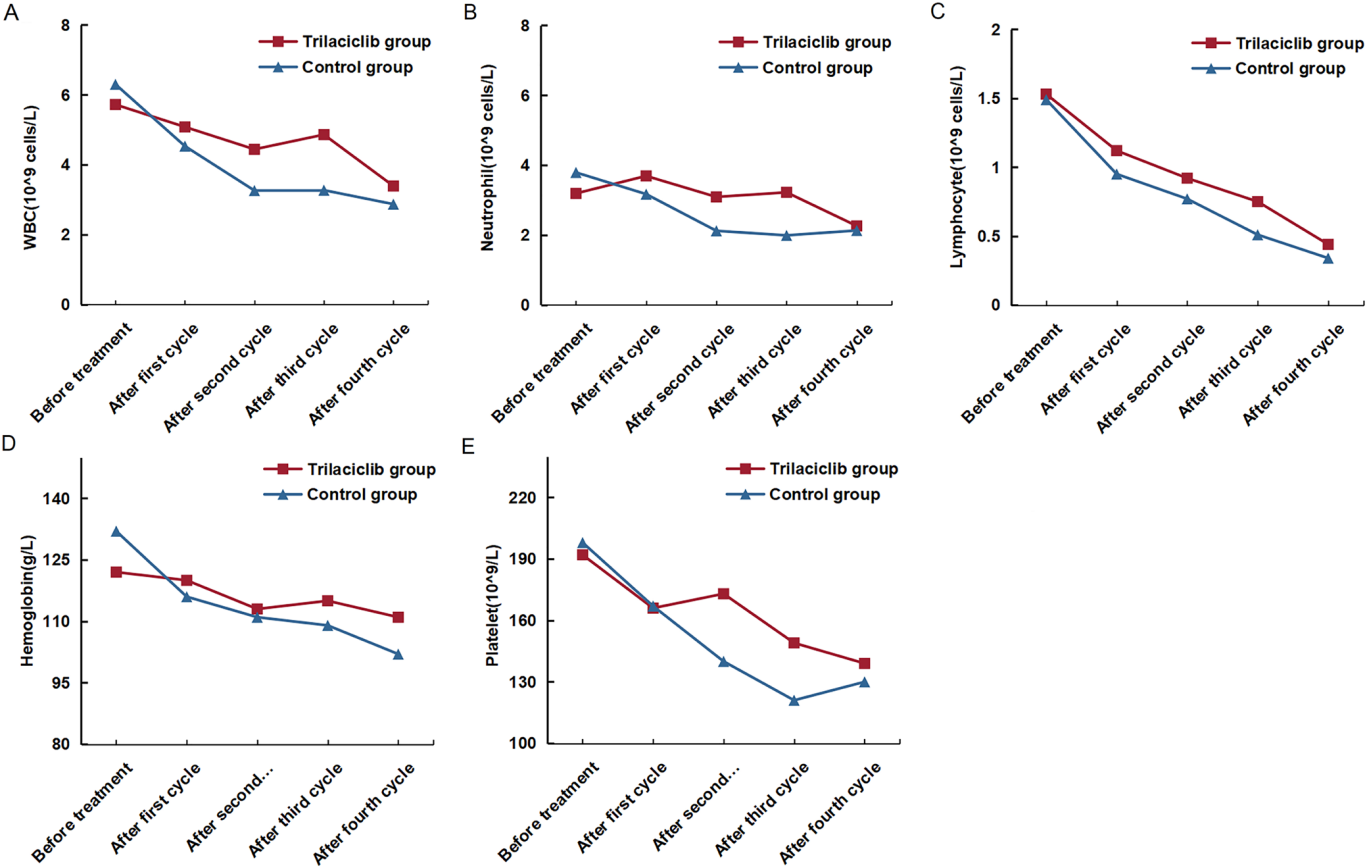
Changes in blood counts during patient treatment. (A) WBC; (B) neutrophil; (C) lymphocyte; (D) hemoglobin; (E) platelet. WBC, white blood cell.

All patients underwent concurrent chemoradiotherapy. All patients, except for three who underwent six cycles of chemotherapy, received four cycles of chemotherapy. As shown in Table 2 and Figure 1, a statistical analysis of the median values of white blood cells, neutrophils, lymphocytes, hemoglobin, and platelets in both patient groups was performed before and after each chemotherapy cycle. As the number of chemotherapy treatments increased, the median values of these blood indicators gradually decreased. However, the degree of decrease in the various blood indicators was less pronounced in the trilaciclib group compared with the control group.

**TABLE 2:** Changes in blood counts during patient treatment (median values).

	WBC (10^9^ cells/L)	Neutrophil (10^9^ cells/L)	Lymphocyte (10^9^ cells/L)	Hemoglobin (g/L)	Platelet (10^9^/L)
Before treatment					
Trilaciclib group	5.72	3.19	1.53	122	192
Control group	6.30	3.79	1.49	132	198
After first cycle					
Trilaciclib group	5.08	3.69	1.12	120	166
Control group	4.53	3.17	0.95	116	167
After second cycle					
Trilaciclib group	4.44	3.09	0.92	113	173
Control group	3.26	2.12	0.77	111	140
After third cycle					
Trilaciclib group	4.86	3.22	0.75	115	149
Control group	3.27	1.99	0.51	109	121
After fourth cycle					
Trilaciclib group	3.39	2.26	0.44	111	139
Control group	2.87	2.13	0.34	102	130

Abbreviation: WBC, white blood cell.

**FIGURE 2:**
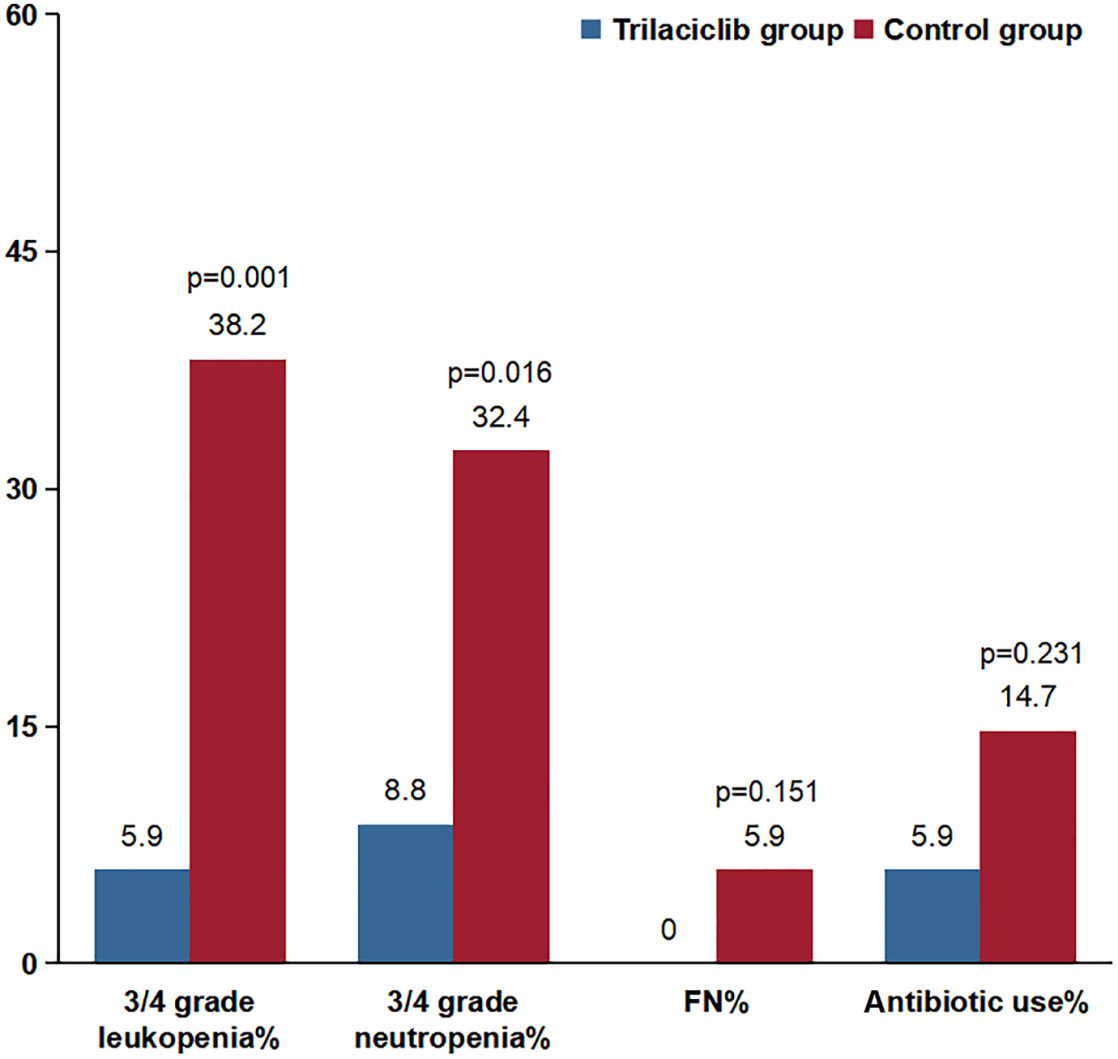
Myeloprotection outcomes associated with trilaciclib and control groups. FN, febrile neutropenia.

Both groups experienced varying degrees of myelosuppression during treatment. None of the patients in the trilaciclib group experienced grade 4 myelosuppression. As shown in Figure 2, the proportion of patients with type III‐IV leukopenia and neutropenia in the trilaciclib group was significantly lower than that in the control group (leukopenia: 5.9% vs. 38.2%, neutropenia: 8.8% vs. 32.4%, all *p* < 0.05). The rate of antibiotic use in the trilaciclib group decreased by 8.8% compared with the control group, suggesting that trilaciclib exerted a protective effect by reducing antibiotic use. None of the patients in the trilaciclib group required antibiotic treatment, whereas two patients in the control group did. Trilaciclib demonstrated a significant protective effect on white blood cells, neutrophils, hemoglobin, and platelets in patients with EC undergoing radiotherapy and chemotherapy (Table 3, all *p* < 0.05). In the Table S1, an analysis of the two groups before PSM showed that the incidence of hematologic adverse events was significantly higher in the control group compared with the trilaciclib group (all *p* < 0.05).

**TABLE 3:** Hematologic TRAEs in patients with propensity score matching.

	Control group (*n* = 34)				Trilaciclib group (*n* = 34)				*p*
	Grade, no. (%)				Grade, no. (%)				
	0–1	2	3	4	0–1	2	3	4	
Leukopenia	5 (14.7%)	16 (47.1%)	12 (35.3%)	1 (2.9%)	23 (67.6%)	9 (26.5%)	2 (5.9%)	0	< 0.001
Neutropenia	13 (38.2%)	10 (29.4%)	7 (20.6%)	4 (11.8%)	28 (82.4%)	3 (8.8%)	3 (8.8%)	0	0.002
Thrombocytopenia	27 (79.4%)	7 (20.6%)	0	0	34 (100%)	0	0	0	0.005
Anemia	22 (64.7%)	11 (32.4%)	1 (2.9%)	0	31 (91.2%)	3 (8.8%)	0	0	0.029

Abbreviation: TRAE, treatment‐related adverse event.

### 3.3 Safety

The non‐hematological toxic reactions in all patients are summarized in Table 4. These toxicities primarily included abnormal transaminases, cough, decreased appetite, nausea, diarrhea, constipation, radiation esophagitis, radiation pneumonia, radiation dermatitis, and abnormal electrolytes. No significant differences were observed in non‐hematological toxic reactions between the trilaciclib and control groups (*p* > 0.05).

**TABLE 4:** Systemic/other TRAEs in patients with propensity score matching.

	Control group (*n* = 34)				Trilaciclib group (*n* = 34)				*p*
	Grade, no. (%)				Grade, no. (%)				
	0–1	2	3	4	0–1	2	3	4	
Fatigue	28 (82.4%)	6 (17.6%)	0	0	26 (76.5%)	8 (23.5%)	0	0	0.549
Decreased appetite	28 (82.4%)	6 (17.6%)	0	0	28 (82.4%)	6 (17.6%)	0	0	1
Hepatic function abnormal	31 (91.2%)	2 (5.9%)	1 (2.9%)	0	33 (97.1%)	1 (2.9%)	0	0	0.498
Renal function abnormal	34 (100%)	0	0	0	33 (97.1%)	1 (2.9%)	0	0	1
Vomiting	31 (91.2%)	3 (8.8%)	0	0	30 (88.2%)	4 (11.8%)	0	0	0.690
Hiccups	32 (94.1%)	2 (5.9%)	0	0	32 (94.1%)	2 (5.9%)	0	0	1
Stomatitis	30 (88.2%)	3 (8.8%)	1 (2.9%)	0	31 (91.2%)	3 (8.8%)	0	0	0.602
Cough	32 (94.1%)	1 (2.9%)	1 (2.9%)	0	32 (94.1%)	2 (5.9%)	0	0	0.513
Constipation	34 (100%)	0	0	0	34 (100%)	0	0	0	1
Diarrhea	32 (94.1%)	2 (5.9%)	0	0	32 (94.1%)	1 (2.9%)	1 (2.9%)	0	0.513
Radiation esophagitis	22 (64.7%)	10 (29.4%)	2 (5.9%)	0	22 (64.7%)	9 (26.5%)	3 (8.8%)	0	0.881
Radiation dermatitis	33 (97.1%)	1 (2.9%)	0	0	31 (91.2%)	3 (8.8%)	0	0	0.303
Radiation pneumonitis	33 (97.1%)	0	1 (2.9%)	0	33 (97.1%)	1 (2.9%)	0	0	0.368
Hypocalcemia	33 (97.1%)	1 (2.9%)	0	0	29	5 (14.7%)	0	0	0.087
Hyponatremia	32 (94.1%)	2 (5.9%)	0	0	32 (94.1%)	2 (5.9%)	0	0	1
Hyperkalemia	33 (97.1%)	1 (2.9%)	0	0	33 (97.1%)	1 (2.9%)	0	0	1
Hypokalemia	26 (76.5%)	7 (20.6%)	1 (2.9%)	0	24 (70.6%)	8 (23.5%)	2 (5.9%)	0	0.787

Abbreviation: TRAE, treatment‐related adverse event.

### 3.4 Response and Survival Outcomes

After PSM, each group consisted of 34 patients (Table 5). In the trilaciclib group, 19 patients achieved CR, 14 achieved PR, and 1 achieved PD. In the control group, 21 patients achieved CR, 11 achieved PR, 1 achieved SD, and 1 achieved PD. The overall response rate (97.1% vs. 94.1%) and DCR (97.1% vs. 97.1%) were similar between the trilaciclib and control groups.

**TABLE 5:** Response rates in patients with propensity score matching.

	Control group (*n* = 34)	Trilaciclib group (*n* = 34)	*p*
CR	21 (61.8%)	19 (55.9%)	0.622
PR	11 (32.4%)	14 (41.2%)	0.451
SD	1 (2.9%)	0	0.314
PD	1 (2.9%)	1 (2.9%)	1
ORR	94.1%	97.1%	0.555
DCR	97.1%	97.1%	1

Abbreviations: CR, complete response; DCR, disease control rate; ORR, objective response rate; PD, progressive disease; PR, partial response; SD, stable disease.

**FIGURE 3:**
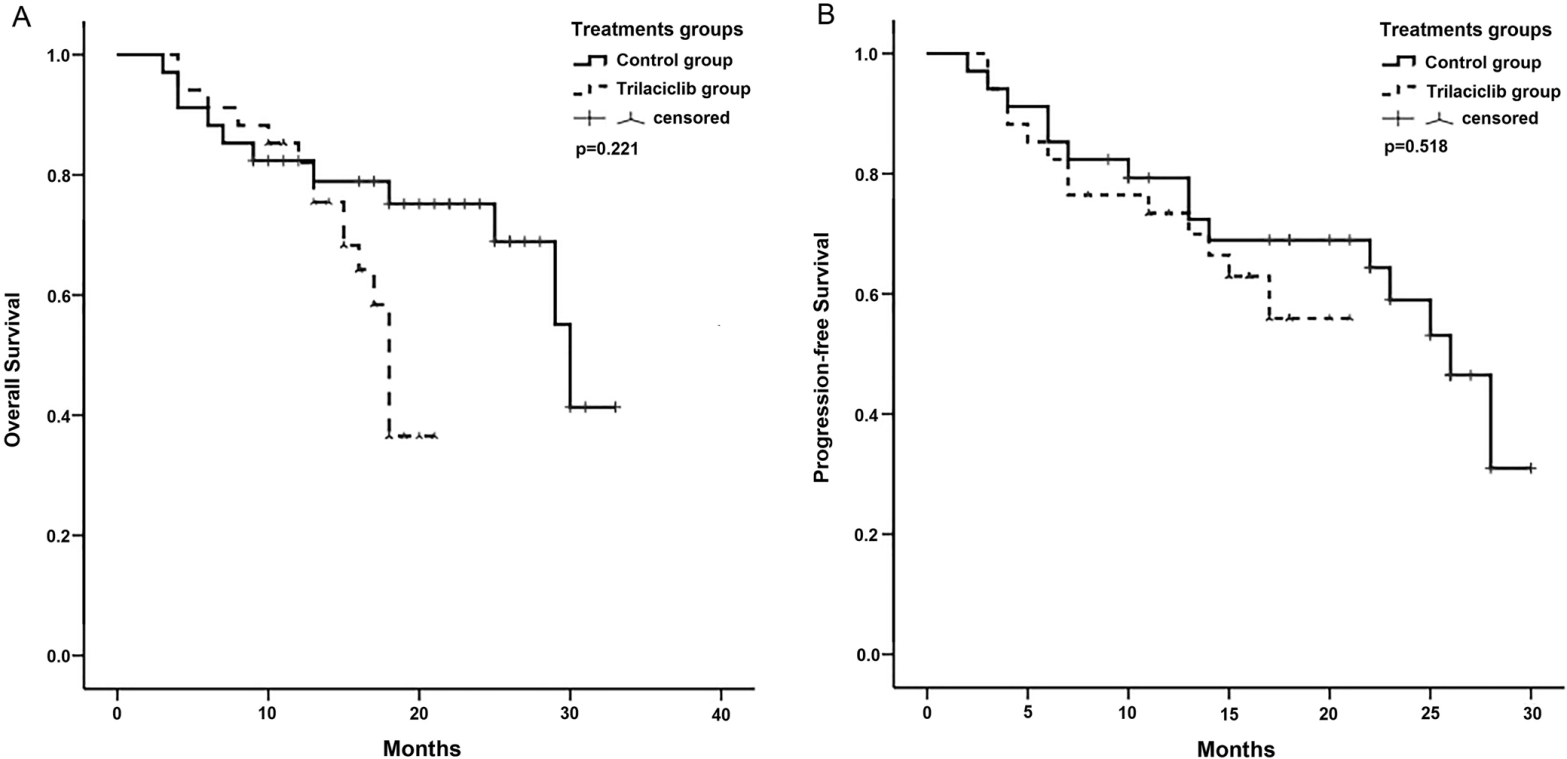
Kaplan–Meier curves of overall survival and progression‐free survival after matching. (A) Overall survival (OS); (B) progression‐free survival (PFS).

The 1‐year OS and PFS rates in the trilaciclib group were 82.0% and 73.4%, respectively. In the control group, the rates were 78.9% and 72.7%, respectively. However, these differences were not significant (Figure 3). The 1‐year PFS rate in the control group before PSM was 67.1%. Although this was slightly lower than that in the trilaciclib group, no significant difference was observed (Figure S1).

## 4 Discussion

EC remains a significant global health concern, with particularly high incidence rates in certain Asian countries, such as China and Iran. Its incidence and mortality rates are expected to increase further in the future [12]. Patients with locally advanced EC who have a favorable overall condition may undergo surgery following neoadjuvant chemoradiotherapy, whereas those with EC who are not suitable for or refuse surgery can receive concurrent chemoradiotherapy. Programmed death‐ligand 1 (PD‐L1) is expressed in approximately 40% of patients with ECs and serves as a dynamic biomarker that can be upregulated following chemoradiotherapy [13]. Consequently, the combination of chemotherapy and immunotherapy has become the standard first‐line treatment for advanced EC. Despite advancements in treatment strategies, chemotherapy remains an effective option for EC management. The primary chemotherapy regimen includes a combination of 5‐fluorouracil, platinum‐based agents, or taxane [14].

Chemotherapy not only eliminates cancer cells but also affects normal, rapidly dividing cells. Its side effects include hematological toxicity and non‐hematological adverse reactions such as nausea, vomiting, and fatigue. Among these, bone marrow suppression is the most common hematological toxicity caused by chemotherapeutic agents. This condition is primarily characterized by a reduction in the number of peripheral blood cells, including white blood cells and neutrophils; thrombocytopenia; and decreased hemoglobin levels. These abnormalities may occur individually or simultaneously [15]. Red bone marrow is the primary hematopoietic organ in adults and is predominantly located at the ends of long, flat, and irregular bones. Radiation exposure induces senescence in bone marrow stromal cells, thereby impairing the ability of hematopoietic stem cells to self‐renew and differentiate. During radiotherapy for EC, the ribs and sternum sustain varying degrees of radiation‐induced damage, with severity influenced by factors such as radiation dose and bone marrow sensitivity [16]. Consequently, patients undergoing concurrent chemoradiotherapy for EC are at a high risk of developing bone marrow suppression. Patients with EC who experience myelosuppression, especially severe cases, exhibit a higher incidence of infection, sepsis, bleeding, and fatigue, frequently resulting in hospitalization. Hematopoietic growth factor support and blood transfusions (red blood cells and/or platelets) are frequently required in such cases. Severe myelosuppression can lead to death [17]. Additionally, bone marrow suppression often necessitates chemotherapy dose reductions and delays in drug administration, thereby compromising therapeutic intensity and antitumor efficacy. Current treatment approaches for bone marrow suppression primarily target a single lineage of blood cells, take a long time to use, require prolonged administration, exhibit delayed efficacy, and pose risks of drug‐ and transfusion‐related adverse reactions. The repeated mobilization of bone marrow hematopoietic stem cells may result in bone marrow exhaustion. Changes in the bone marrow hematopoietic function can compromise the body's tolerance to treatment, adversely affecting the prognosis and therapeutic outcomes in patients with cancer [18]. Research findings suggest that among patients with locally advanced EC who received neoadjuvant concurrent chemoradiotherapy, the 5‐year OS is significantly lower than in those with severe myelosuppression compared with those without myelosuppression (15.4% vs. 69.0%, *p* = 0.003) [19].

Trilaciclib is a highly potent, selective, and reversible CDK4/6 inhibitor administered intravenously prior to chemotherapy to preserve hematopoietic stem and progenitor cells (HSPCs) and enhance antitumor efficacy. The proliferation of HSPCs and lymphocyte populations relies on CDK4/6 activity and is arrested in the G1 phase of the cell cycle after exposure to trilaciclib [20, 21, 22, 23, 24]. This transient, drug‐induced cell cycle arrest caused by trilaciclib prevents HSPC proliferation in the presence of cytotoxic drugs, thereby mitigating chemotherapy‐induced cell damage. When combined with immunotherapy, trilaciclib can modulate the tumor immune microenvironment through transient T‐cell suppression [8, 25, 26]. The G1T28‐04 study conducted in patients with advanced triple‐negative breast cancer (TNBC) [27] revealed that although the duration and incidence of grade 4 neutropenia did not show significant improvement in the trilaciclib‐chemotherapy combination group, a substantial enhancement in OS was observed. The median OS of patients with advanced TNBC improved (19.8 months vs. 12.6 months, *p* < 0.0001), with a 63% reduction in the mortality risk. Notably, a greater benefit was observed in the PD‐L1‐positive population (PD‐L1‐positive: 32.7 months vs. 10.5 months, hazard ratio (HR)s = 0.34; PD‐L1‐negative: 17.8 months vs. 13.9 months, HR = 0.48).

Currently, clinical research data on trilaciclib has primarily focused on patients with small‐cell lung cancer and TNBC. Three randomized, double‐blind, Phase II clinical trials were conducted in patients with SCLC [28]: G1T28‐02, G1T28‐05, and G1T28‐03. These clinical trials demonstrated that the administration of trilaciclib prior to chemotherapy can reduce the duration of severe neutropenia in the first cycle from 4 to 0 days, reduce the incidence of severe neutropenia by 78.4% (52.9%–11.4%, *p* < 0.001), the incidence of grade 3/4 anemia by 31.9% to 20.3% (*p* = 0.0279), and the incidence of grade 3/4 thrombocytopenia by 36.1% to 19.5% (*p* = 0.0067). Based on these findings, trilaciclib was recognized as a breakthrough therapy in the United States in August 2019. In February 2021, it was approved for use in patients with SCLC undergoing treatment with a platinum‐containing combination of etoposide or a topotecan‐containing regimen. In July 2022, trilaciclib was approved for marketing in China for patients with extensive‐stage SCLC who had not previously received systemic chemotherapy. However, no clinical data on its use in the treatment of EC in combination with chemoradiotherapy remain unavailable.

Our center included 34 patients with EC who received trilaciclib for synchronous chemoradiotherapy prior to the full course of chemotherapy. These patients were matched with 34 individuals with clinical characteristics comparable to those in the control group through PSM analysis. The degree of decline in all hematological parameters was significantly lower in the trilaciclib group compared with the control group, particularly in terms of the preservation of white blood cells, neutrophils, and platelets. These findings confirmed the substantial bone marrow protective effect of trilaciclib (Table 2 and Figure 1). As illustrated in Figure 1, the median values of white blood cells and neutrophils in the control group following the fourth cycle of chemotherapy were comparable to those observed in the trilaciclib group. This outcome may be attributed to the administration of long‐acting white blood cell injections after severe bone marrow suppression in the control group. Additionally, trilaciclib demonstrated a protective effect on lymphocytes. The G1T28‐04 study [27] revealed that CD^8+^ T cells in the trilaciclib group exhibited higher levels of interferon production compared with the control group, suggesting that trilaciclib may enhance T‐cell activation and improve the efficacy of immunotherapy. Preclinical research on trilaciclib indicates that short‐term exposure to trilaciclib induces alterations in tumor gene expression, increasing the expression of genes associated with tumor immunity while downregulating immunosuppressive gene expression. Additionally, trilaciclib regulates the composition of immune cells within tumors, attenuates the function of immunosuppressive Treg cells, and enhances the proportion of cytotoxic T lymphocytes. Trilaciclib exhibits a synergistic effect when combined with PD‐L1 inhibitors and chemotherapy, demonstrating the potential to prolong survival in murine models [26, 29, 30]. In terms of clinical efficacy, the ORR and DCR in the trilaciclib group were comparable to those in the control group. Moreover, no significant differences were observed in the 1‐year OS and PFS between the two groups. Preclinical data have shown that in tumor‐bearing mouse models that are either sensitive or insensitive to CDK4/6 inhibitors, trilaciclib does not interfere with the inhibitory effects of chemotherapy on tumor growth [31]. The G1T28‐04 clinical study conducted in patients with TNBC demonstrated that trilaciclib exhibits comparable efficacy regardless of CDK4/6 dependency, indicating that it does not interfere with the cytotoxic effects of chemotherapy on tumor cells [32]. Furthermore, multiple clinical studies have confirmed that trilaciclib does not increase the safety risk of chemotherapeutic drugs, and the overall incidence of adverse events is lower than that of chemotherapy alone. Consistently, our study found no significant difference in the incidence of other toxic side effects between the trilaciclib and control groups.

This study has some limitations. First, as a real‐world retrospective analysis, its findings require validation through a prospective randomized controlled trial to further confirm the bone marrow‐protective effects of trilaciclib in EC. Our center has obtained ethical approval for this prospective clinical study and is currently enrolling patients. Second, due to the recent market introduction and the high cost of trilaciclib as an innovative drug, the sample sizes of both groups were small. However, data analysis still demonstrated the strong bone marrow‐protective effect of trilaciclib. Third, as the study population consisted exclusively of Chinese patients with esophageal squamous cell carcinoma, the findings may not be generalizable to a broader population. In addition to the limitations of the study, trilaciclib has some disadvantages compared with traditional myelosuppressive agents, such as G‐CSF and recombinant erythropoietin. Although trilaciclib has the potential to prevent myelosuppression, this benefit is not entirely unique. A previous study reported that pegylated recombinant human G‐CSF, when administered before chemotherapy, can also prevent neutropenia [33]. As a novel drug, trilaciclib has limited use in the clinical setting, and the lack of sufficient safety studies presents a notable challenge. Additionally, traditional myelosuppressive drugs can directly stimulate bone marrow hematopoiesis, leading to a more rapid and effective response in patients with myelosuppression.

In summary, although symptomatic treatments for chemotherapy‐induced bone marrow suppression exist, the available options are limited, each presenting additional risks. Reducing the incidence of bone marrow suppression is essential to improving patients' quality of life, enhancing treatment compliance, preventing treatment interruptions, and ensuring therapeutic efficacy. The findings of this study showed that the administration of trilaciclib before chemotherapy in patients with EC undergoing synchronous chemoradiotherapy reduced the incidence of bone marrow suppression while maintaining a favorable safety profile. These results provide a foundation for the potential application of trilaciclib in the treatment of other tumor types in clinical practice.

## 5 Conclusion

Trilaciclib effectively prevented myelosuppression in patients with EC undergoing concurrent chemoradiotherapy, demonstrating a favorable safety profile and therapeutic efficacy.

## Author Contributions

**Jingze Yan:** data curation (equal), writing – original draft (equal), writing – review and editing (equal). **Zeyuan Liu:** data curation (equal), formal analysis (lead). **Hui Chen:** funding acquisition (equal), investigation (equal). **Xinchen Sun:** investigation (equal), writing – review and editing (equal). **Xiaolin Ge:** project administration (equal), supervision (equal). **Xiaojie Xia:** conceptualization (equal), writing – review and editing (equal).

## Ethics Statement

The study was conducted in accordance with the Declaration of Helsinki. The Ethics Committee of the First Affiliated Hospital of Nanjing Medical University approved the study protocol and the registration number is 2024‐SR‐313.

## Conflicts of Interest

The authors declare no conflicts of interest.

## Supporting information


Data S1.

## Data Availability

The data sets and materials in this study are available from the corresponding author.
