# Supplementary material for: Real‐World Assessment of Trilaciclib for the Prevention of Chemoradiotherapy‐Induced Myelosuppression in Esophageal Squamous Cell Carcinoma: A Propensity Score Matching Study
Source: Cancer Med. 2025 Apr 15;14(8):e70862. doi: 10.1002/cam4.70862 (PMC11998604; doi:10.1002/cam4.70862)

Supplementary Table. Hematologic TRAEs in patients before propensity score matching.

|  | Control group (n=169) | | | | Trilaciclib group (n=34) | | | | p-value |
| --- | --- | --- | --- | --- | --- | --- | --- | --- | --- |
|  | Grade, No.(%) | | | | Grade, No.(%) | | | |  |
|  | 0-1 | 2 | 3 | 4 | 0-1 | 2 | 3 | 4 |  |
| Leukopenia | 30 (17.7%) | 71 (42.0%) | 61 (36.1%) | 7  (4.3%) | 23 (67.6%) | 9 (26.5%) | 2 (5.9%) | 0 | ＜0.001 |
| Neutropenia | 65 (38.5%) | 56  (33.1%) | 32 (18.9%) | 16 (9.4%) | 28 (82.4%) | 3 (8.8%) | 3 (8.8%) | 0 | ＜0.001 |
| Thrombocytopenia | 126 (74.5%) | 26 (15.4%) | 13 (7.7%) | 4 (2.3%) | 34 (100%) | 0 | 0 | 0 | 0.012 |
| Anemia | 101  (59.8%) | 57 (33.8%) | 9 (5.4%) | 2  (1.2%) | 31 (91.2%) | 3 (8.8%) | 0 | 0 | 0.006 |

TRAE: treatment-related adverse event.

Supplementary Figure. Kaplan–Meier curve of progression-free survival before propensity score matching.


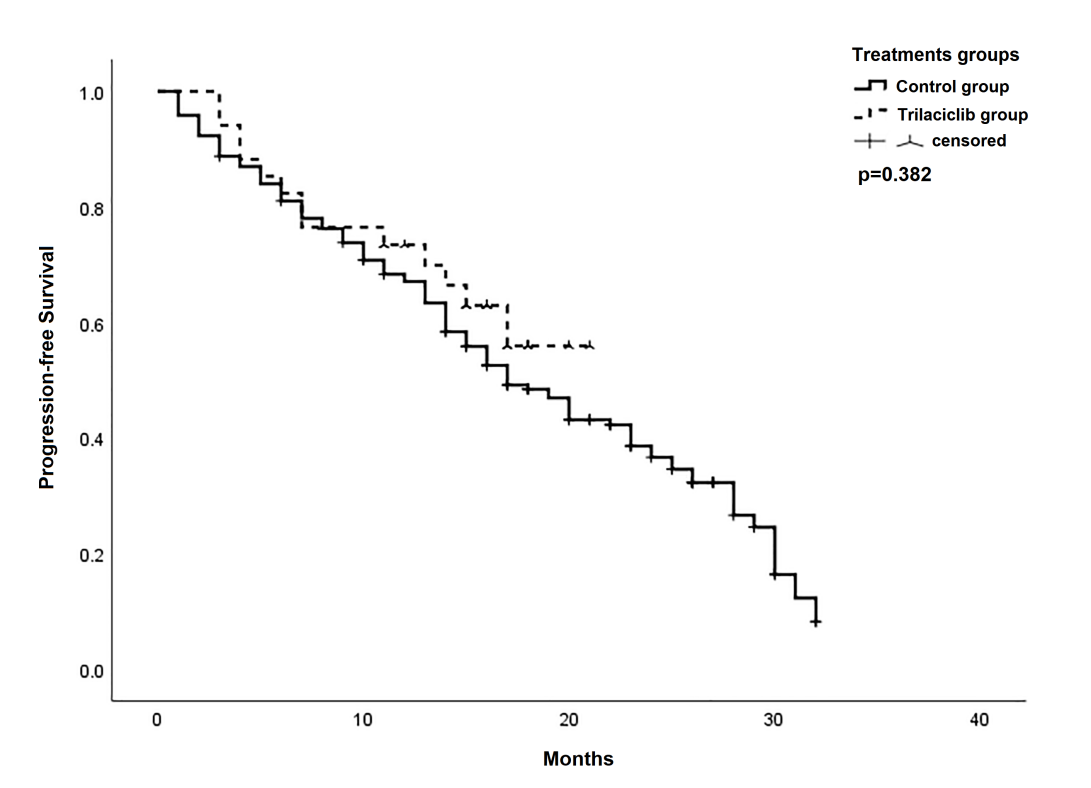

Supplement: Supplementary file 1 — Data S1. [file CAM4-14-e70862-s001.docx]
